# Diagnostic value of the microcolon using ultrasonography in small bowel atresia

**DOI:** 10.1186/s12887-022-03629-z

**Published:** 2022-10-06

**Authors:** Hao Ju, Shu Feng, Ying Huang

**Affiliations:** Department of Ultrasound, Shengjing Hospital of China Medical University, 110004 Shenyang, China

**Keywords:** Neonate, Intestinal atresia, Intestinal obstruction, Ultrasonography, Small bowel atresia

## Abstract

**Background:**

Microcolon helps diagnose small bowel atresia (SBA) using contrast enema. However, there are no ultrasonography (US) microcolon criteria for diagnosing SBA. Therefore, this study aimed to evaluate colon accuracy and other characteristics for diagnosing SBA by US, using surgical or clinical information as the reference standard.

**Methods:**

US was performed on 46 neonates aged ≤ 7 days old. In the study group (n = 15), neonates with SBA were confirmed following surgery. In the study group without SBA (n = 15), neonates with other gastrointestinal problems besides SBA were confirmed by surgical or clinical follow-up. Sixteen neonates without gastrointestinal problems were classified as the control group. The colonic diameter was measured, and colonic gas was sought and observed. Statistical analysis was performed to compare US parameters between the study group and other two groups. The optimal cut-off value of the colonic diameter for SBA diagnosis was obtained using receiver operating characteristic analysis.

**Results:**

Colonic diameters (0.5 cm) in the study group (interquartile ranges [IQR], 0.5–0.6 cm) was significantly smaller than that in the group without SBA (0.9 cm; IQR, 0.8–1.2 cm) (*P <*  0.001) and in the control group (1.2 cm; IQR, 0.8–1.35 cm) (*P <* 0.001). Optimum cut-off value for diagnosing SBA was 0.65 cm (sensitivity, 90.3%; specificity, 86.7%; accuracy, 89.1%) for the colonic diameter. Combining microcolon and gas-negativity showed the best performance in SBA diagnosis using US, with increased accuracy (91.3%).

**Conclusion:**

A colon < 0.65 cm in diameter should be called a microcolon; combining US with gas-negativity is an essential diagnostic basis for SBA.

**Supplementary Information:**

The online version contains supplementary material available at 10.1186/s12887-022-03629-z.

## Background

Congenital small bowel atresia (SBA), including duodenal, jejunal, and ileal atresia, is a common cause of neonatal intestinal obstruction, with an incidence ranging from 1.3 to 2.8 of 10,000 live births [1]. Prenatal ultrasound can be used to diagnose intestinal obstruction, but it can only play a role of preliminary screening for SBA, as dilated bowel loops and polyhydramnios provide a low overall detection rate [2, 3]. Neonates with SBA need further examination for diagnosis after birth. Diagnosing neonatal SBA primarily depends on clinical manifestations and imageological examination. Plain abdominal radiographs can be used to investigate intestinal obstruction. Upper gastrointestinal contrast studies can identify obstruction levels and rule out volvulus and malrotation diseases [4]. A contrast enema helps investigate suspected Hirschsprung disease [5]. Although barium and water-soluble enema can detect microcolon and facilitate SBA diagnosis, the radiation and risk of intestinal perforation have led many scholars to recommend minimising their use [6–9]. Ultrasonography (US), a safe, convenient and inexpensive imaging method, is suitable for neonates because of their thin abdominal wall. The microcolon without gas, significantly smaller than a normal colon, can be used to diagnose SBA by US [10]. However, no standard colonic measurement is available for new-borns because of the lack of consistency in US scans. Moreover, there is no standard diametric value for diagnosing SBA, which limits the diagnostic ability of clinical sonographers. A better US method for differentiating patients with SBA from those without SBA could reduce the need for contrast enema and upper gastrointestinal contrast. Therefore, this study aimed to evaluate colon accuracy and other characteristics for diagnosing SBA by US, using surgical or clinical information as the reference standard.

## Methods

### Patients

**Fig. 1:**
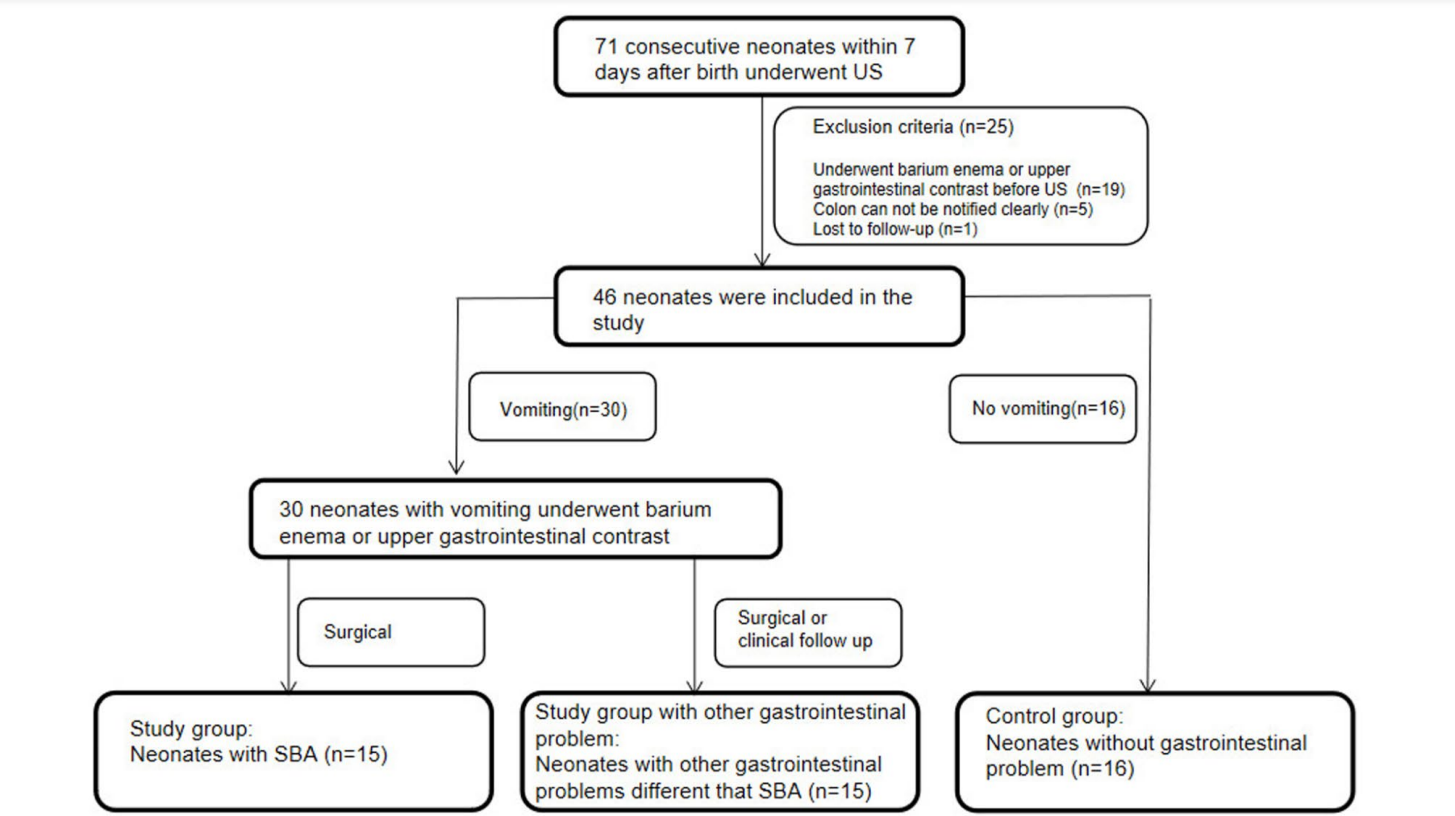
Flow diagram of inclusion criteria of neonates

Institutional review board approval was obtained for this study. The need for informed consent was waived because our examination was performed as a routine abdominal US. Between July 2019 and October 2021, 71 consecutive neonates aged ≤ 7 days underwent US in our hospital’s ultrasound department (Fig. 1). Of these, 19 neonates were excluded because they underwent upper gastrointestinal or contrast enema before US. Furthermore, we excluded five neonates whose colon could not be identified clearly using ultrasound and one who was lost to follow-up. Consequently, 46 neonates were included in our study (male-to-female ratio, 25:21; preterm-to-term ratio, 20:26). Thirty neonates who were vomiting underwent upper gastrointestinal contrast or contrast enema after the ultrasound examination. As a study group, SBA was confirmed at surgery in 15 vomiting cases (male-to-female ratio, 7:8; preterm-to-term ratio, 5:10), including duodenal (n = 2), jejunal (n = 7), and ileal atresia (n = 6). In the group without SBA, 15 vomiting cases with other gastrointestinal illnesses besides SBA were confirmed with surgical or clinical follow up (male-to-female ratio, 9:6; preterm-to-term ratio, 5:10), including annular pancreas (n = 5), intestinal stenosis (n = 3), anal atresia (n = 1), congenital megacolon (n = 2), gastroesophageal reflux (n = 1), and neonatal vomiting (n = 3), according to surgery or upper gastrointestinal contrast. The remaining 16 cases without gastrointestinal problems were classified as the control group (male-to-female ratio, 9:7; preterm-to-term ratio, 10:6). We recorded their postnatal time, and clinical follow-up was carried out until they were 1 month old (Table 1).

**Table 1:** Clinical data findings of patients with study group and other groups

Variable	Study group(n = 15)	Study group without SBA(n = 15)	Control group(n = 16)	***P*** Value
Male-to-female ratio	7:8	9:6	9:7	0.715^†^, 0.724^‡^
Preterm-to-term ratio	5:10	5:10	10:6	1.000^†^, 0.156^‡^
Patient age (days)^*^	1 (0.71, 2 ), (0.58-7)	1 (0.63, 3), (0.54-4)	2 (1, 4.5) (0.16-6)	0.3129
Clinical feature	duodenal atresia (n = 2), jejunal atresia (n = 7), ileal atresia (n = 6)	annular pancreas (n = 5), intestinal stenosis (n = 3), anal atresia (n = 1), congenital megacolon (n = 2), gastroesophageal reflux (n = 1), neonatal vomiting (n = 3)	jaundice (n = 12) exomphalos (n = 4)	

Study group, SBA neonates;

Study group without SBA, neonates with gastrointestinal problems different that SBA;

Control group, neonates without gastrointestinal disease

*Data are median (quartile), followed with the ranges in parentheses

† The *P* value concerns statistical comparison between study group and study group without SBA. The χ^2^ test was used for statistical comparison

‡ The *P* value concerns statistical comparison between study group and control groups. The χ^2^ test was used for statistical comparison.

### US techniques

**Fig. 2:**
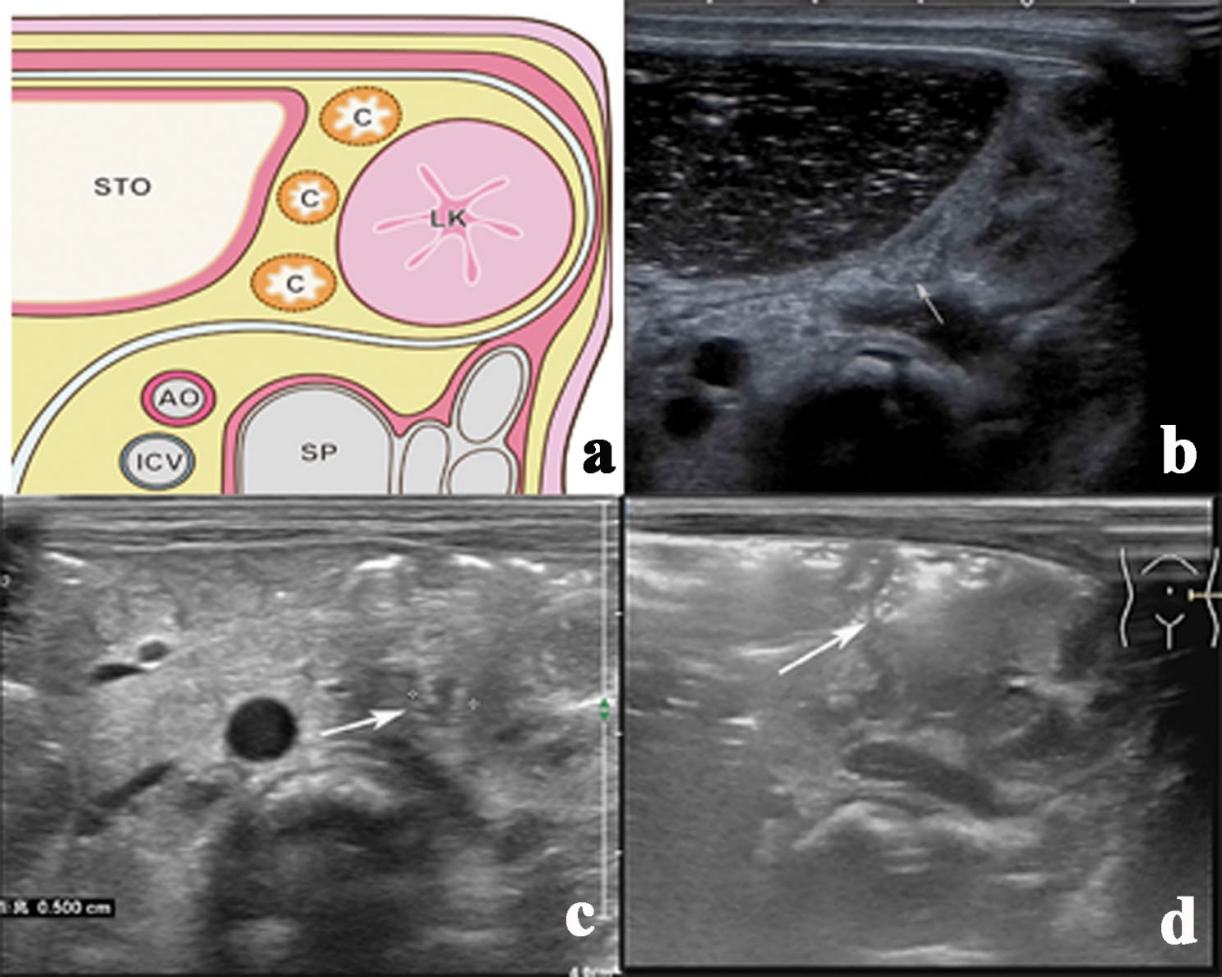
Transection US images of the colon. **(a)** Schematic diagram of colon position on ultrasound. The letter ‘C’ in the lumen represents the likely location of the colon. Sto: stomach, SP: spine, LK: left kidney, AO: abdominal aorta, ICV: inferior cava vena **(b)** descending colon (arrow) without gas in a 2-day-old female neonate with SBA. The colon is small and without gas. **(c)** descending colon (arrow) without gas in a 1-day-old female neonate with an annular pancreas. There is little fluid and no gas in the colon. **(d)** descending colon (arrow) with gas in a 2-day-old female healthy neonate. There is some fluid and a little gas in the colon

**Fig. 3:**
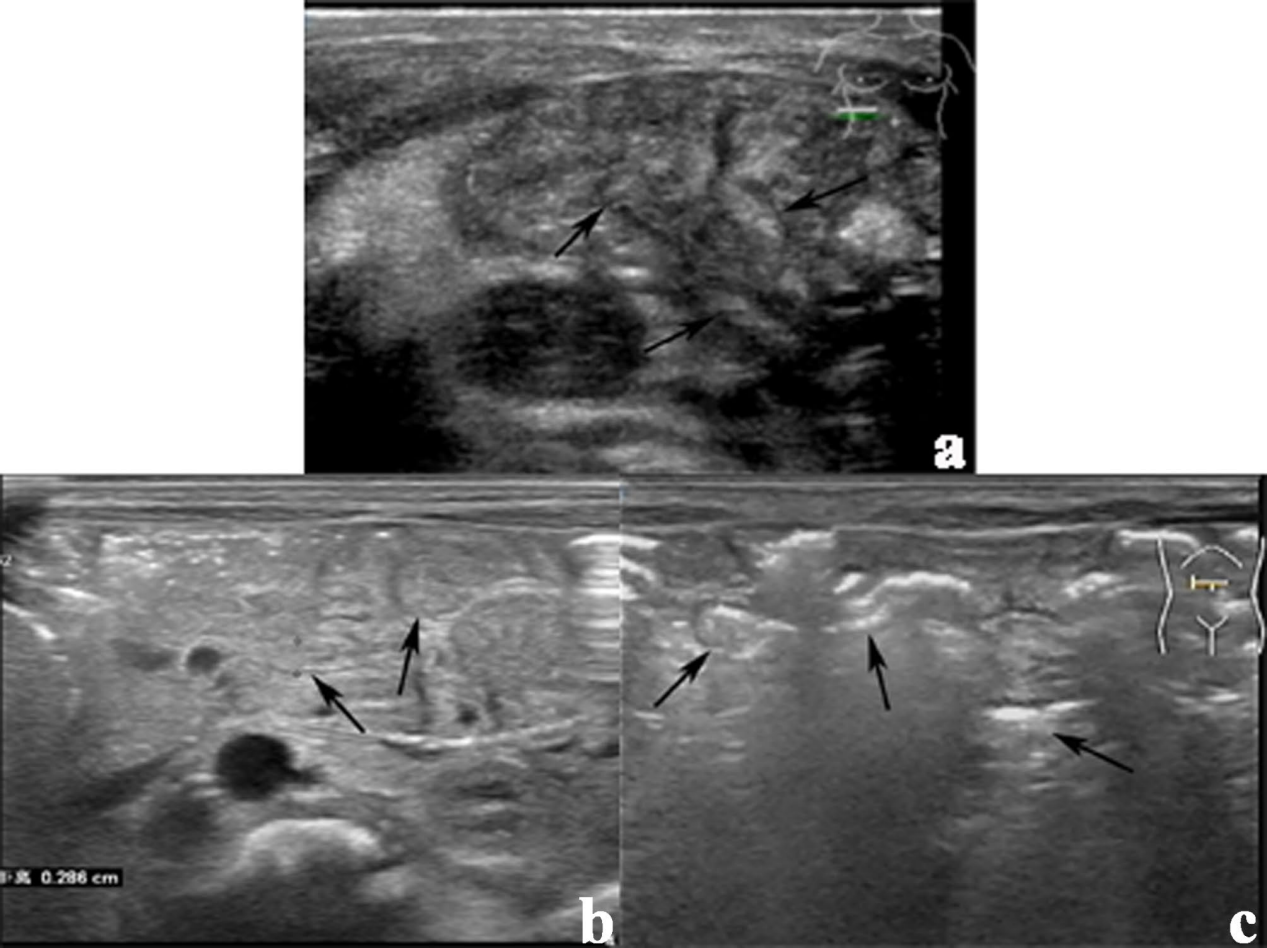
US shows the small bowel. **(a)** Gas negative in the small bowel. A 1-day-old female neonate with SBA. The micro small bowel has no gas or content (arrow). **(b)** Gas negative in the small bowel. A 1-day-old female neonate with an annular pancreas. The micro small bowel has no gas or content (arrow). **(c)** Gas in the small bowel. A 2-day-old male healthy neonate. So much gas in the micro small bowel (arrow)

Neonates underwent US using 3–9 MHz (Philips iU22, Philips Healthcare, Bothell, WA, USA) and 5–12 MHz transducers (Philips EPIQ7C, Philips Healthcare, Bothell, WA, USA). They were kept quiet by making them suck on a pacifier while the examinations were carried out. We measured the colon diameter at the left kidney’s transverse level (Fig. 2). We also measured the diameter of the smallest small bowel of the abdomen. Subsequently, we evaluated the presence of micro small bowel and the presence of gas in the colon and small bowel. The micro small bowel was defined as the small bowel diameter < 0.6 cm. Gas-negative colon was defined as the absence of gas in the measured colon (Video 1). A gas-negative small bowel was defined as the absence of gas in the micro small bowel on an ultrasound image (Fig. 3) (Video 2). US was performed by one operator with > 10 years of experience with paediatric US. Two other paediatric radiologists with > 5 years of experience performed all measurements and evaluations offline. Differences in opinions were resolved through discussions, when necessary.

### Statistical analysis

Data are presented as medians and interquartile ranges (IQRs). The nonparametric Wilcoxon test for paired data was used to test the difference between the study group and the other groups. The chi-squared and fisher exact tests were used to assess categoric data (neonatal sex, preterm or term, presence of micro small bowel, gas in small bowel and colon or not). The kruskal-wallis test was used to test the difference of ages between three groups. Receiver operating characteristic (ROC) curves were constructed to assess colon diameter and yielded the most accurate SBA diagnosis. Fifteen neonates with SBA and 31 without SBA were included in the ROC analysis. The optimal cut-off points for SBA prediction were identified from the highest Youden index. Areas under the ROC curves with 95% confidence intervals were calculated for the colon diameter. The cut-off value’s sensitivity and specificity for the colon diameter were calculated. We evaluated the sensitivities and specificities of other US SBA abdominal findings (presence of micro small bowel and the presence or absence of gas in the small bowel and colon), in combination with the preceding criteria for colon diameter, to assess the diagnostic accuracy added with measurement of the colon size. Statistical analysis was performed with IBM SPSS version 26.0 (IBM Corp. New York, USA). A two-tailed *P*-value < 0.05 indicates a statistically significant difference.

## Results

### US abdominal findings

**Fig. 4:**
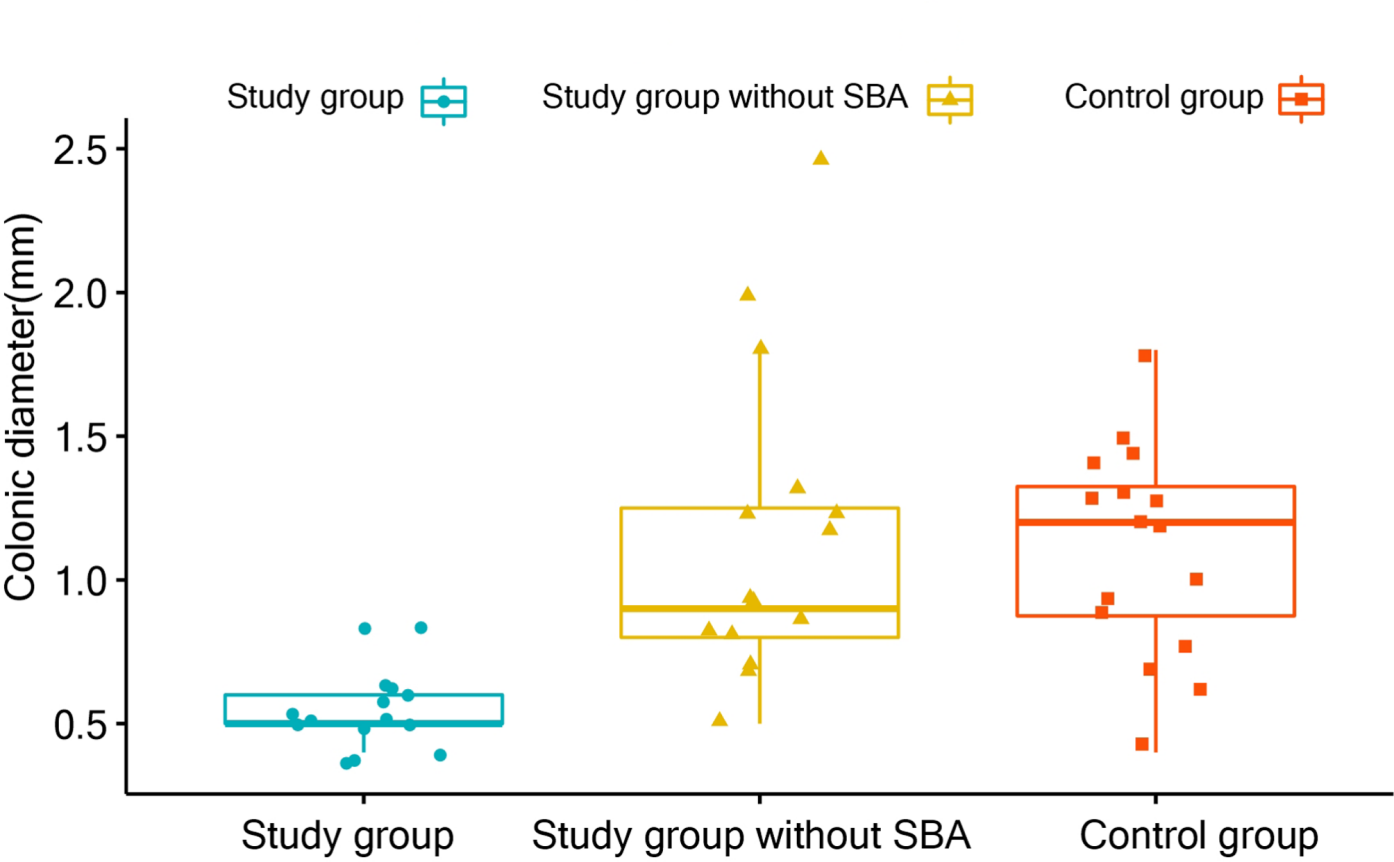
The nonparametric Wilcoxon test for paired data was used to test the difference in colonic diameter between the study group and the other groups

Clinical characteristics (including preterm or term, male or female, and ages) between the study group and other groups showed no significant difference (*P* > 0.05) (Table 1). The colonic diameter in the study group of 0.5 cm (IQR, 0.5–0.6 cm) was significantly smaller than that in the group without SBA (0.9 cm; IQR, 0.8–1.2 cm; *P* <  0.001) and in the control group (1.2 cm; IQR, 0.8–1.35 cm; *P* <  0.001) (Fig. 4). The micro small bowel was found in all 15 neonates with SBA; there was no gas in the micro small bowel and colon for all of them. Among neonates in the group without SBA, the micro small bowel was visualised in 80.0% (12 of 15), including annular pancreas (n = 5), intestinal stenosis (n = 3), congenital megacolon (n = 1), gastroesophageal reflux (n = 1), and neonatal vomiting (n = 2). The proportion of gas-negativity in the small bowel was approximately 40.0% (6 of 15), including annular pancreas (n = 2), intestinal stenosis (n = 2), congenital megacolon (n = 1), and neonatal vomiting (n = 1). The proportion of gas-negativity in the colon was approximately 26.0% (4 of 15), including annular pancreas (n = 1), intestinal stenosis (n = 2), and anal atresia (n = 1). In the control group, the micro small bowel was noticeable in 68.8% (11 of 16); there were gas-negative small bowels in 25.0% (4 of 16) of the cases. Only one neonate, aged 4 h postnatal (the youngest), had a gas-negative colon. There was no significant difference in the presence of micro small bowel between the study group and the group without SBA (*P* = 0.224). Neonates in the study group had more micro small bowels than those in the control group (*P* =  0.043). There were significant differences in the gas-negative colon and small bowel between the study group and other groups (*P* <  0.001); neonates in the study group had less gas in the colon and distal small bowel (Table 2).

**Table 2:** Comparison of US findings of small bowel and colon in the three groups

US Finding	Study group ( n = 15 )	Study group without SBA ( n = 15 )	Control group ( n = 16 )	***P*** value
Colon diameter (IQR)	0.5 cm (0.5–0.6 cm)	0.9 cm (0.8-1.2 cm)	1.2 cm (0.8-1.35 cm)	< 0.001^†^; 0.001^‡^
Micro small bowel (<0.6 cm)	15 (100%)	12 (80%)	11 (68.8%)	0.224^†^; 0.043^‡^
Gas negative in small bowel	15 (100%)	6 (40%)	4 (25%)	< 0.001^†^; 0.001^‡^
Gas negative in colon	15 (100%)	4 (26.0%)	1 (6.3%)	< 0.001^†^; 0.001^‡^

IQR, interquartile ranges

† The *P* value concerns statistical comparison between study group and study group without SBA. The χ^2^ test was used for statistical comparison

‡ The *P* value concerns statistical comparison between study group and control group. The χ^2^ test was used for statistical comparison

### Cut-off value and accuracy

**Fig. 5:**
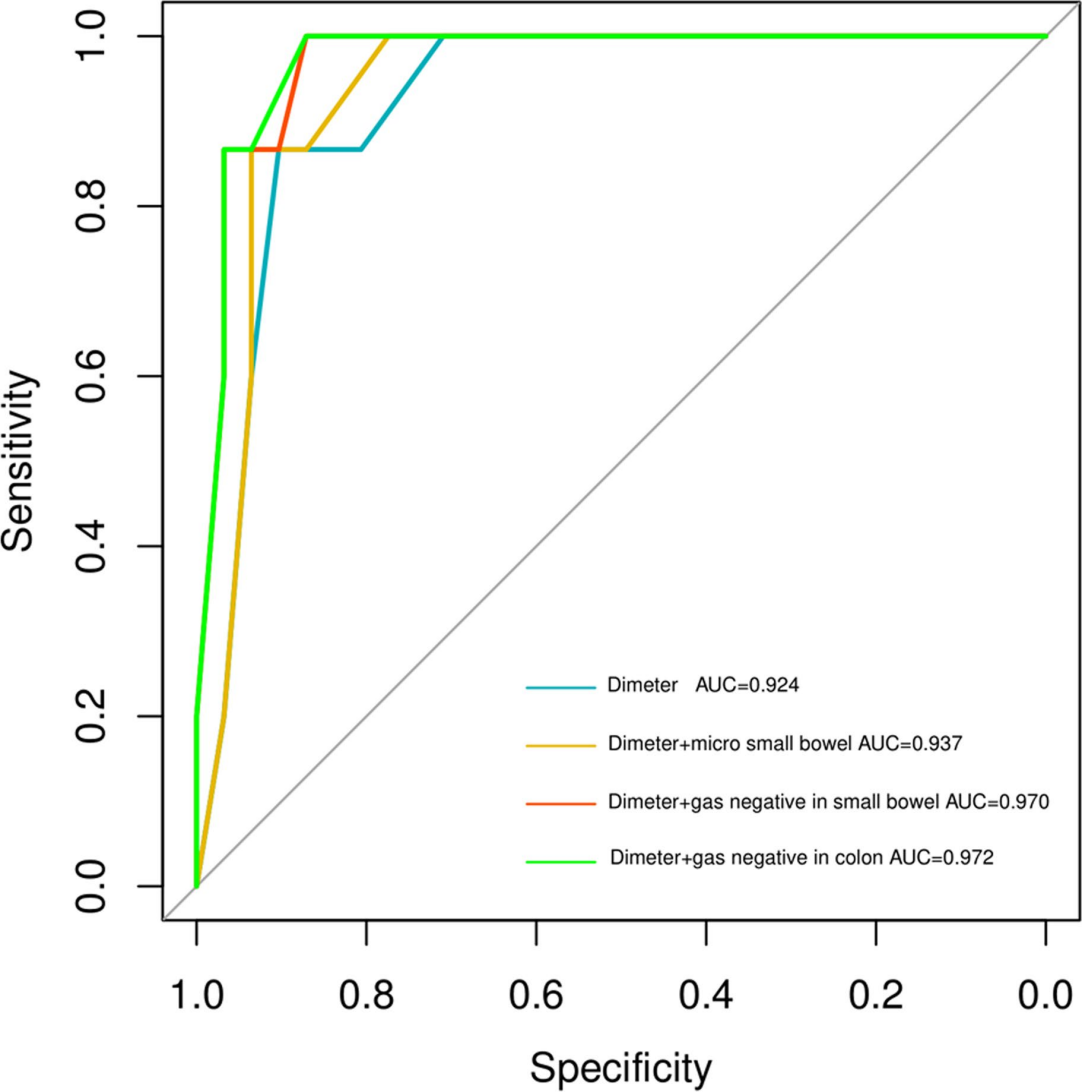
Using ROC analysis to find a combination of colon diameter with the other criteria

According to the ROC analysis, the optimal cut-off value of the colon was defined as 0.65 cm (area under the ROC curve, 0.924). This criterion showed 90.3% (28 of 31) sensitivity, 86.7% (13 of 15) specificity, and 89.1% (41 of 46) accuracy. When the colonic diameter was 0.85 cm, the criterion showed a 71.0% (22 of 31) sensitivity and a 100% (15 of 15) specificity. Combining the colonic diameter and the micro small bowel produced a sensitivity of 93.5% (29 of 31), a specificity of 86.7% (13 of 15), and an accuracy of 91.3% (42 of 46). Combining the colon diameter and gas-negativity in the small bowel showed a sensitivity of 87.1% (27 of 31), a specificity of 100% (15 of 15), and an accuracy of 91.3% (42 of 46) (Table 3). Combining the colonic diameter and gas-negativity provided a sensitivity of 93.5% (27 of 31), a specificity of 100% (15 of 15), and an accuracy of 91.3% (42 of 46). Combining the colon diameter and colonic gas-negativity showed the best performance in SBA US diagnosis, with the highest area under the curve (AUC) (Fig. 5).

**Table 3:** Diagnostic performance of the combination of each US findings for SBA.

US Finding of abdomen	True-Positive Findings in Patients with SBA (n = 15)	True-Negative Findings in Neonate Without SBA (n = 31)	Sensitivity (%)	Specificity (%)	Accuracy (%)	95% CI	AUC
Colon diameter	13	28	0.903	0.867	0.8913	0.8466-1	0.9237
Colon diameter and micro small bowel	13	29	0.935	0.867	0.9130	0.8648-1	0.9366
Colon diameter and gas negative in small bowel	15	27	0.871	1.0	0.91304	0.9268-1	0.9699
Colon diameter and gas negative in colon	15	27	0.871	1.0	0.91304	0.9305-1	0.9720

CI, confidence interval; AUC, area under the curve

## Discussion

Only a few ultrasound studies have focused on the neonatal intestine, possibly because it can be interfered by intestinal gas and stool mass. However, many intestinal diseases can be diagnosed by US even with interference [11–14]. US has become an important diagnostic method in many children’s diseases, including intussusception [15], intestinal malrotation [16, 17], and intestinal polyp [18]. US is important to neonates in diagnosing necrotising enterocolitis [19]. The proximal intestine is often dilated for neonates with SBA, filling with amniotic fluid or milk. In contrast, to avoid aspiration and relieve pain, clinicians usually carry out gastrointestinal decompression for neonates when an intestinal obstruction is suspected, which leads to the production of more intestinal gas, relieves the dilation state, and disturbs doctors from finding the dilated bowel. The distal SBA intestine was not affected by swallowing and gastrointestinal decompression. Since no amniotic fluid and milk were passed, the distal intestine (including the colon and a part of the small bowel) remained in a state of reduction for a long time. It only has a little meconium content and is without any gas. These characteristics are easily detected on ultrasound and can be used to distinguish other types of intestinal obstruction. Briefly, the present study examined the distal small bowel and colon characteristics to determine the accuracy of the SBA diagnosis.

The microcolon has been recognised and proposed by surgeons for nearly 100 years [20]. It has now become an important basis for contrast enema in diagnosing SBA; however, it was barely mentioned on the US. Although Hao [10] reviewed the ultrasonographic findings of 19 neonates with SBA and confirmed that US could detect microcolon without gas, the study did not provide the criteria of microcolon and the data on false-negative and -positive rates. To ensure consistency, we selected a transverse view of the left kidney showing the short colonic axis for measurement. This area was chosen because the anatomic locations of the descending colon are more constant and hardly disturbed by intestinal gas at the specified section. Finding a colon requires some experience and patience. Gentle pressure is applied, if necessary, to avoid the influence of crying. Furthermore, there is a need to avoid treating dilated small bowel as colon. In this study, five neonates had an unclear or unexplored colon. In addition to the high display rate (93.0%, 67/71), the colonic diameter of the study group was significantly smaller than that of the other two groups, indicating that the colonic diameter has a more intuitive value in SBA diagnosis. Combined with the cut-off value of the ROC curve, we believe that the ultrasonic colon with a diameter of < 0.65 cm has the maximum predictive value for SBA, called ultrasonic microcolon. On ultrasound, SBA can be ruled out when the colon diameter is > 0.85 cm.

We postulated that distal micro small bowel might be a secondary change of the SBA due to prolonged complete obstruction. But it is difficult to assess and make repeatable measurements. Because these distal micro small bowel perform small, peristalsis slower, have no fixed location, and are often obscured by dilated intestines. For atresia of or near the ileocecal junction, the micro small bowel may be absent or difficult to find [7]. We performed a periumbilical scan to find the section with the smallest intestine and the least intestinal gas for analysis. The diameter was < 0.6 cm and without wriggle, defined as the micro small bowel. Moreover, all neonates with SBA not only had micro small bowel but were also gas-negative. However, the micro small bowel was also present in the study group without SBA and the control group. It may be because the neonates have not accumulated food in the intestinal tract soon after birth. In particular, some neonates with heavy intestinal stenosis in the study group without SBA have a high proportion of micro small bowel.

Gas-negativity in the distal intestine is an essential diagnostic basis for SBA. Excluding few individuals has a biliary fistula [21]. In the study group without SBA, neonates without colonic gas were mostly patients with severe intestinal obstruction. In the control group, there was only one case of a 4-hour-old new-born, the youngest neonate, with a gas-negative colon. The gas in the intestinal lumen had not moved to the colon because the neonate did not eat much after birth. This shows that the absence of gas in the colon could be physiological during the first 4 h of their life. Since the amount of gas can be changed over time, finding a micro-small bowel without gas requires more experience, it cannot be an independent SBA diagnosis. Similarly, we did not find a time point for detecting false-positive gas negativities in the small bowel.

The current findings do not justify using colonic ultrasound as a substitute for contrast enema in diagnosing SBA; however, our study provides clinicians and neonates with more options. For neonates with suspected SBA, US can be done first; if SBA can be diagnosed, contrast enema administration may be cancelled. The difference in diagnostic accuracy between US and contrast enema requires further clarification.

Our study had some limitations. Firstly, although our study spanned 2 years, we did not have enough cases, including the control group of normal new-borns, because they rarely received routine abdominal ultrasounds. More clinical data need to be accumulated for comparison in differentiating colonic diameter between SBA and other diseases, including meconium ileus, even if no case was gathered in this study. Secondly, it is challenging to determine accurately the amount of micro small bowels, and the amount of gas in it can change over time. The results may vary depending on the doctor’s experience. Thirdly, considering the benefits of timely gastrointestinal decompression, we could not require eliminating gastrointestinal decompression in suspicious SBA neonates before US. Therefore, proximal bowel dilation was not analysed in this study. However, contrast enema or upper gastrointestinal contrast cannot be performed before an ultrasound examination is still required.

## Conclusion

A colon < 0.65 cm in diameter should be called a microcolon; combining US combined with gas-negativity is an essential diagnostic basis for SBA.

## Electronic supplementary material

Below is the link to the electronic supplementary material.

Supplementary Material 1

Supplementary Material 2

Supplementary Material 3

## Data Availability

All data generated or analysed during this study are included in this published article and its supplementary information files.

## Abbreviations

SBA: small bowel atresia
US: ultrasonography
IQR: interquartile ranges
ROC: receiver operating characteristic
