# Supplementary material for: Diagnostic value of the microcolon using ultrasonography in small bowel atresia
Source: BMC Pediatr. 2022 Oct 6;22:576. doi: 10.1186/s12887-022-03629-z (PMC9535889; doi:10.1186/s12887-022-03629-z)
Supplement: Supplementary file 3 — Supplementary Material 3 [file 12887_2022_3629_MOESM3_ESM.doc]

We want to publish the videos with the submission.

**Video_1** Microcolon without gas, surrounding by some dilating small bowel.

**Video_2** Micro small bowel without gas, surrounding by some dilating small bowel.
